# The Early Intervention of a Class III Malocclusion With an Anterior Crossbite Using Chincup Therapy: A Case Report

**DOI:** 10.7759/cureus.62473

**Published:** 2024-06-16

**Authors:** Shefali Singh, Rizwan Gilani, Anjali Kathade, Aishwarya R Atey, Srushti Atole, Pratik Rathod

**Affiliations:** 1 Department of Orthodontics and Dentofacial Orthopedics, Sharad Pawar Dental College and Hospital, Datta Meghe Institute of Higher Education and Research, Wardha, IND; 2 Department of Conservative Dentistry and Endodontics, Sharad Pawar Dental College and Hospital, Datta Meghe Institute of Higher Education and Research, Wardha, IND

**Keywords:** orthodontics, pseudo class iii, anterior crossbite, class iii malocclusion, chincup

## Abstract

Class III malocclusions with anterior crossbites pose significant challenges in orthodontic treatment, especially in growing children. This case report details the early intervention of a 12-year-old patient presenting with a Class III malocclusion characterized by an anterior crossbite and mandibular prognathism. A chincup was employed to inhibit mandibular growth and encourage maxillary development. Consistent use of the chincup, with regular follow-ups and adjustments, led to significant improvements. The anterior crossbite was corrected, resulting in a Class I molar relationship and an improved facial profile. The maxillary arch perimeter increased, providing space for the eruption of canines and premolars. This case demonstrates that early intervention with a chincup can effectively manage Class III malocclusion with an anterior crossbite, highlighting the importance of timely orthodontic assessment and treatment to achieve stable, long-term results.

## Introduction

Three main types of Class III malocclusions are recognized: skeletal, dental, and pseudo-Class III malocclusions. In 1966, Tweed was the first to establish a classification scheme for Class III malocclusions, which separated the cases into groups: pseudo and skeletal Class III. The feature of pseudo-Class III malocclusion is an anterior crossbite resulting from the functional shift of the mandible [1].

Maxillary hypoplasia, maxillary retrognathism, pure mandibular prognathism (MP), or a combination of the above two are the causes of Class III malocclusion [1]. This kind of malocclusion might affect one or both jaws in terms of sagittal length or location in relation to one another, this suggests the possible anatomical heterogeneity of this condition. Furthermore, research implies environmental and inherited variables may play a major role in the development of Class III malocclusion [2]. Class III malocclusion has been linked to a number of environmental factors, including habits, enlarged tonsils, aberrant tongue and mandibular position, endocrine problems, nasal blockage, and prolonged mouth breathing that causes the jaw to develop downward and backward [2]. However, there are not many observations to support the significance of these elements.

For orthodontists, treating MP or skeletal Class III malocclusion in developing youngsters is a substantial problem. For skeletal Class III instances, a number of therapeutic strategies have been devised. These include the Fränkel functional regulator III appliance, maxillary protraction in conjunction with chincup traction, face masks, and chincup traction [1]. For growing patients with a somewhat protruding jaw, a reasonably normal anteroposterior posture, and a normal maxilla size, the chincup is usually advised. Chincup therapy has been used for MP for a long time, although results vary. This could be because of a number of factors, including the patient's age, the force of the appliance, and the length of treatment [3].

In cases of late deciduous or early mixed dentition, the chincup is especially useful for treating MP [3]. After the intermaxillary skeletal imbalance is resolved, the appliance is changed to a retainer for the balance of the treatment period by reducing the force applied to the chincup and the amount of time it is used [4].

Treatment plans for adults are based on how severe the malocclusion is. Camouflage is a compensatory orthodontic treatment that modifies the position of the teeth relative to their supporting bone, to disguise underlying jaw discrepancies [4]. It can be used to manage mild Class III malocclusion with an acceptable facial profile. Achieving appropriate occlusion, aesthetics, and functionality is the aim. Orthognathic surgery is necessary for severe instances with an undesirable facial profile. It may entail mandibular setback, maxillary advancement, or a combination of the two. Usually, a traditional orthodontic therapy phase comes before one of these surgical treatments. The use of extraction to conceal a skeletal malocclusion gained popularity in the 1930s and 1940s when growth modification was still considered largely ineffectual, and surgical correction was still in its infancy [2]. This marked the introduction of camouflage treatment within orthodontics. Although it might not address the skeletal issue or facial profile, the proclination of the maxillary incisors and retroclination of the mandibular incisors is typically the method used to disguise a Class III malocclusion and enhance overall occlusion [5].

## Case presentation

A 12-year-old male patient reported to the Department of Orthodontics and Dentofacial Orthopedics at Sharad Pawar Dental College and Hospital, Wardha, India, with a chief complaint of backwardly placed upper front teeth.

**Figure 1:**
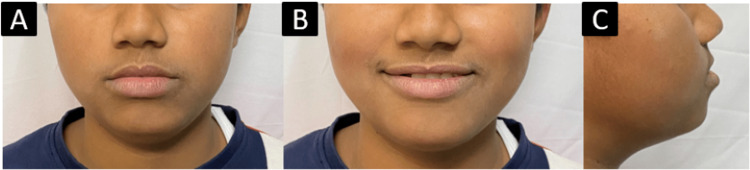
Pretreatment extraoral photographs: A) frontal; B) smiling; C) profile

Extraoral examination revealed that the patient had an apparently symmetrical face with a mesoprosopic face form. The patient had competent lips with shallow mentolabial sulcus. The smile of the patient was symmetrical and consonant (Figure 1).

**Figure 2:**
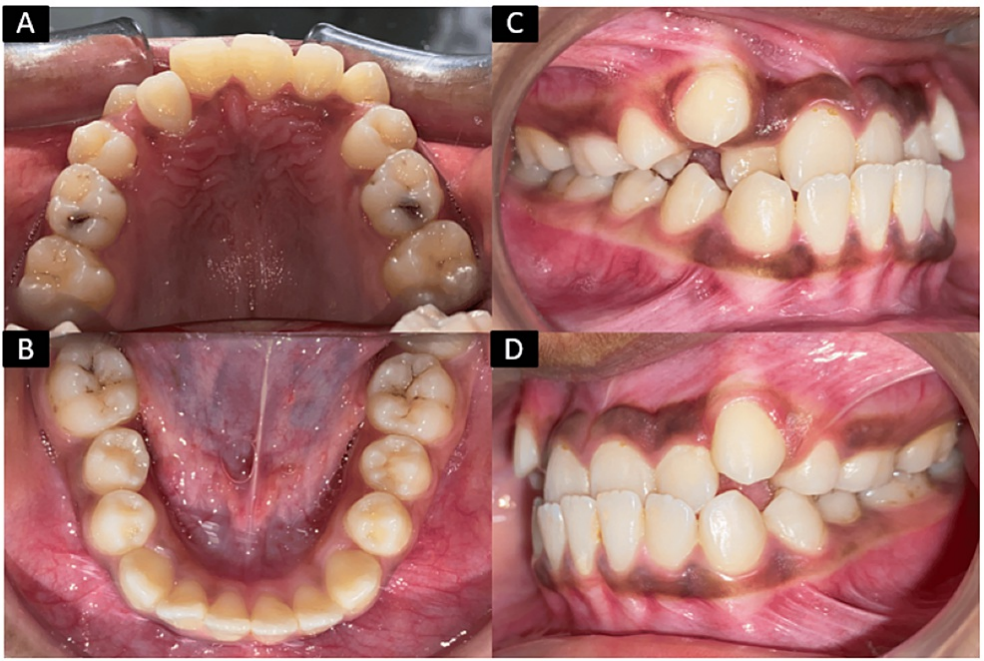
Pretreatment intraoral photographs: A) maxillary arch; B) mandibular arch; C) right occlusion; D) left occlusion

On intraoral examination, a mixed dentition was observed. An adequate zone of attached gingiva was present with satisfactory gingival health. All permanent teeth had erupted except the second premolars and third molars in all four quadrants. The deciduous second molar (E) was present on both sides of the upper arch. Upright incisors were seen in the upper arch, with anterior crowding and upper midline shift towards the left side. Anterior crossbite was seen with tooth numbers 11, 12, 21, and 22. Both maxillary and mandibular arches were U-shaped with Class I molar and canine relationship on both sides. Negative overjet and overbite were 2 mm and 3 mm, respectively. Upper canines were buccally erupted (Figure 2).

Functional examination showed normal speech pattern, oro-nasal breathing, and matured swallowing pattern. The mandible closure path was deviated towards the left side, and there were no associated signs and symptoms of temporomandibular disease.

**Figure 3:**
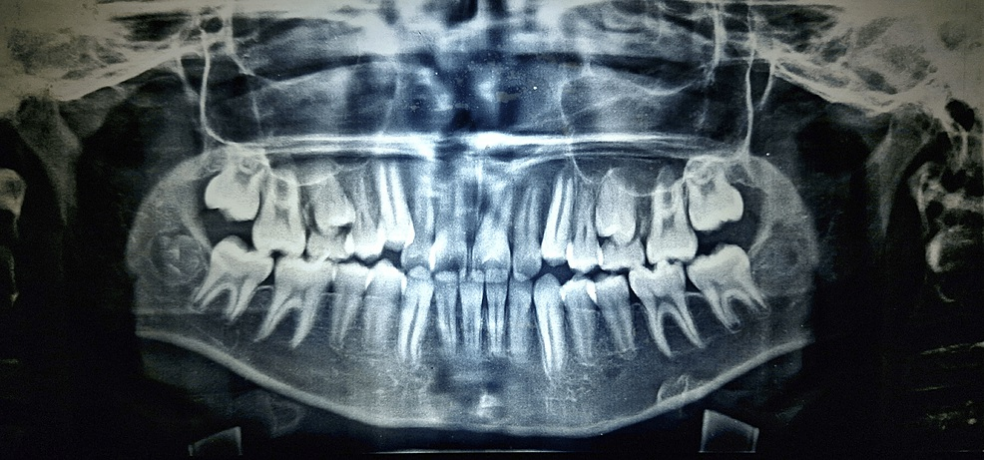
Pretreatment orthopantomogram showing the presence of all teeth except third molars, and the presence of deciduous molars in the first and second quadrants

Orthopantomogram (OPG) examination revealed the presence of all teeth in all four quadrants, except the upper and lower third molars. The roots of the second premolars were yet to be completed (Figure 3).

**Figure 4:**
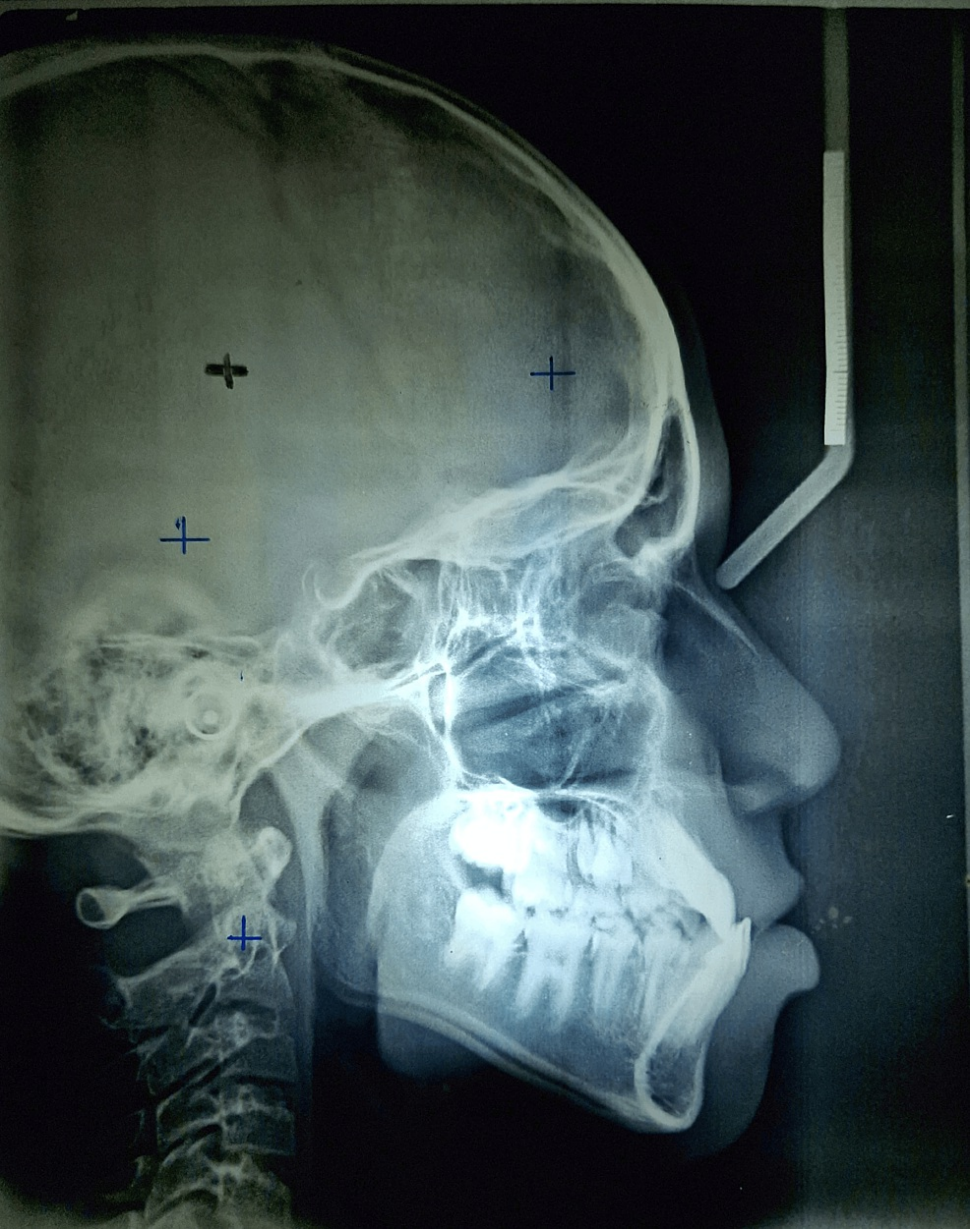
Pretreatment lateral cephalogram showing CVMI stage II (acceleration), anterior crossbite, and Class III skeletal pattern CVMI: cervical vertebrae maturation index

The patient was in cervical vertebrae maturation index (CVMI) stage II (acceleration) with Class III skeletal bases (beta angle: 42°) and a vertical growth pattern (Frankfort mandibular plane angle (FMPA): 27°) revealed in cephalometric analysis. Soft tissue analysis revealed competent lips with an obtuse nasolabial angle (Figure 4).

Orthodontic phase

**Figure 5:**
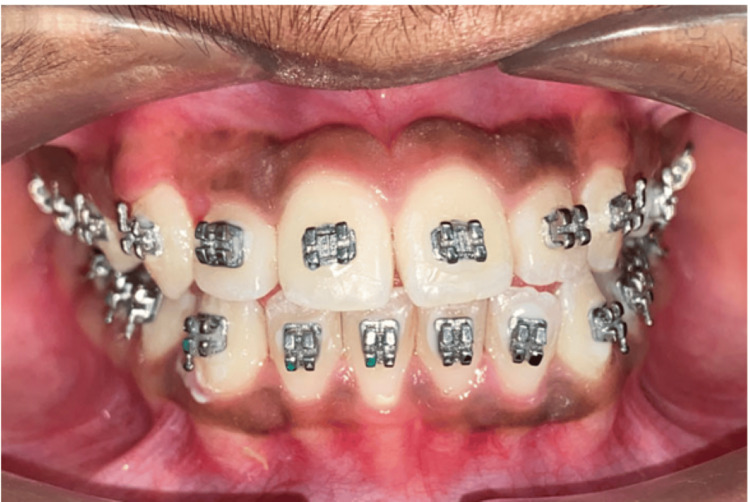
Mid-treatment photograph after correction of anterior crossbite

The bonding of the upper anterior teeth was done using a McLaughlin, Bennett, and Trevisi (MBT) 0.022” slot and banding of the upper first molars was done. A 0.014” NiTi wire was given in the upper arch, only engaging the anterior teeth, and the bite was raised. Using this arrangement (2 × 4 appliance), the anterior crossbite was corrected. Later, after the exfoliation of the upper second deciduous molars, the entire upper arch was bonded, including erupting teeth 15 and 25, and leveling and alignment of the upper arch was completed. Along with the alignment of the upper arch, the lower arch was also bonded and aligned simultaneously (Figure 5).

**Figure 6:**
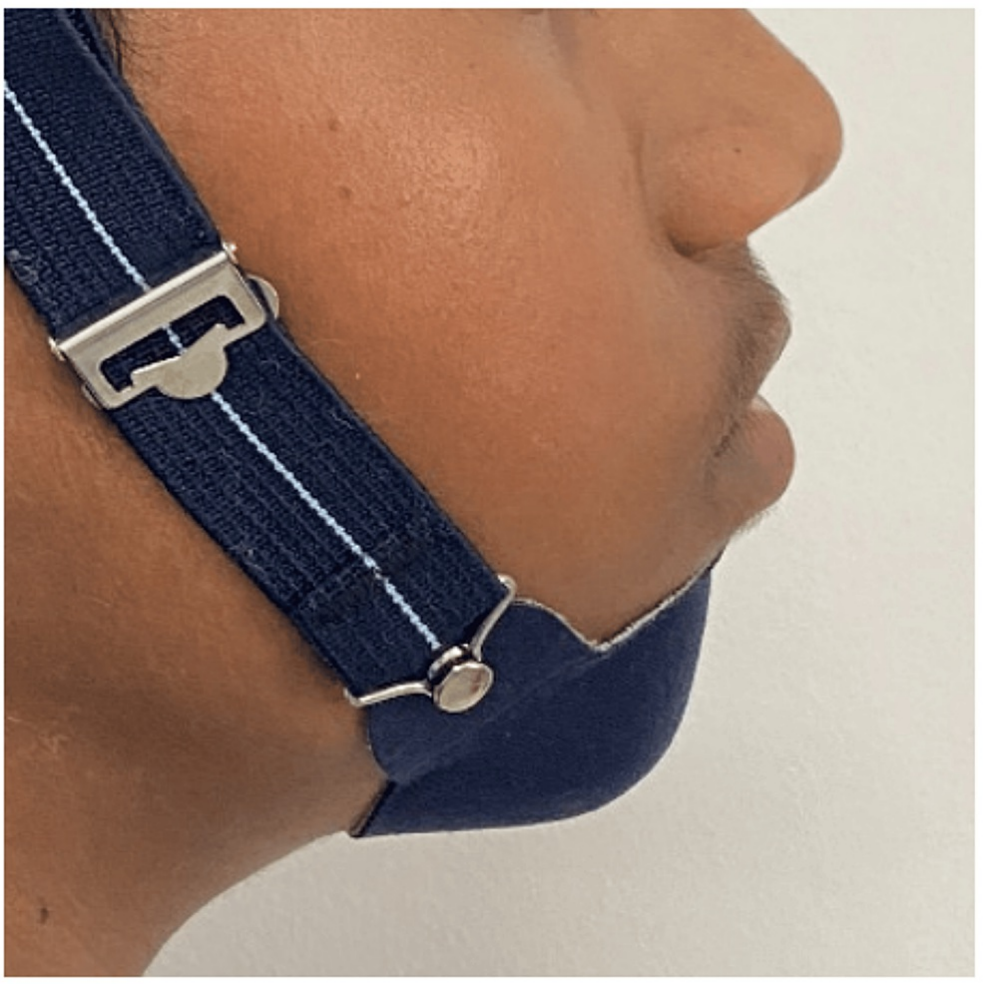
Extraoral photograph with chincup appliance

In order to limit mandibular growth, the patient was instructed to wear a medium-sized chincup appliance for at least 14 hours a day, which applied 250-300 gm force from start to end of the treatment, in conjunction with the orthodontic therapy (Figure 6).

Results

**Figure 7:**
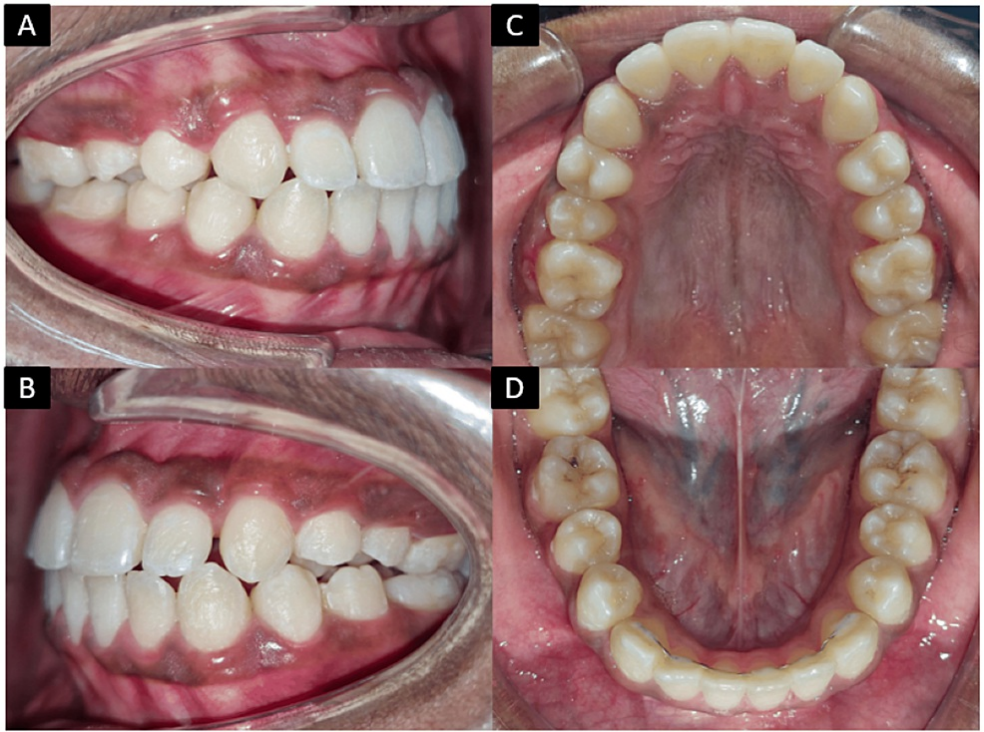
Post-treatment intraoral photographs: A) right occlusion; B) left occlusion; C) maxillary arch; D) mandibular arch

After 25 months of treatment, significant orthodontic objectives were successfully achieved. The upper and lower teeth were properly aligned, including the previously buccally placed upper canines. Anterior crossbite was corrected, resulting in the establishment of a normal overjet and overbite. Additionally, a Class I relationship for both canines and molars was attained, contributing to an overall improvement in dental alignment (Figure 7).

**Figure 8:**
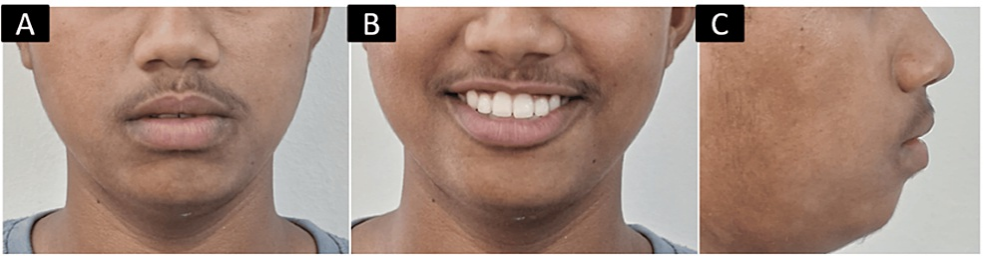
Post-treatment extraoral photographs: A) frontal; B) smiling; C) profile

Furthermore, the patient's concave profile was corrected, enhancing their facial aesthetics (Figure 8).

## Discussion

An anterior crossbite is indicative of pseudo-Class III malocclusion, which arises from a functional displacement of the mandible in a forward direction [2]. These patients usually show a mesial step of less than 3 mm in mixed dentition, with mandibular incisors being proclined and spaced, while maxillary incisors are frequently retroclined. Patients with functional Class III malocclusion typically exhibit an edge-to-edge incisor connection with a shift of mandible in foreword, when directed into a centric relationship. This is mostly because of the maxillary incisor retroclination [6].

The Class I molar relationship may conceal underlying skeletal abnormalities in these patients, despite a straight facial profile and normal appearance of the mandible. Facemasks worn for short bursts of time, detachable appliances, and partially fixed appliances are a few treatment options for minor skeletal Class III malocclusion and anterior crossbite in mixed dentition [6]. To optimize the orthopedic effects and guarantee treatment stability, early anterior crossbite repair is essential [1]. Furthermore, the maxillary arch perimeter is increased when an anterior crossbite is corrected, providing more room for premolar and canine eruption [7].

Mandibular functional shifts can happen in a number of ways. Unilateral posterior crossbites are caused by lateral functional shifts, whereas pseudo-Class III malocclusions are caused by forward functional shifts. There are two primary causes of forward functional shifts, which are commonly linked to both anterior and posterior crossbites [8].

First, some higher anterior teeth that erupt lingually interact with lower teeth due to occlusal interference, which prevents correct mandibular closure in the centric relation. Second, the mandibular arch is wider posteriorly than the maxillary arch, which leads to the development of the posterior crossbite [4]. A posterior crossbite occurs when the mandible moves forward, causing its broader posterior arch to occlude with the straight frontal section of the maxilla [8].

By opening the jaw, the neuromuscular reaction conditioned by the presence of occlusal interferences is eliminated, correcting the forward functional shift of the mandible. This method is in line with research, according to Adly et al., which showed that bite opening caused all kinds of functional shifts to vanish, enabling the mandible to automatically return to its normal functional position, even before occlusal interferences were eliminated [9].

If left untreated, anterior crossbites can cause disruption in the growth pattern, and incisal and gingival wear of teeth present in the lower anterior region. In order to cure Class III malocclusions, removable functional appliances like Frankel III regulators and activators allow maxillary molar eruption, while keeping mandibular molars in their proper positions. This causes an occlusal plane rotation, which changes the Class III to Class I molar relation.

When using a facemask for maxillary protraction, the maxilla rotates anticlockwise and the mandible rotates clockwise, which usually resulting a lower face height increment. Hence, patients having functional Class III with lower angles are more suited for these gadgets. Because maxillary expansion promotes forward and downward migration of the maxilla and improves orthopedic stability, it can improve outcomes for young children with anterior crossbite [9]. In about 84% of cases, self-correction is expected to occur without the need for extra appliances. When maxillary expansion is combined with fixed appliances, the arch perimeter is also improved, which lessens the need for extractions in patients who have mild to moderate crowding. Up to 6.0 mm, more can be added to the maxillary arch perimeter with this enlargement. Fixed appliances offer other benefits, such as the application of light continuous forces and better three-dimensional control of tooth movement [10].

## Conclusions

The management of Class III malocclusions, particularly in growing children, presents significant challenges but offers various effective treatment options. Understanding the distinct characteristics and complications associated with different types of Class III malocclusions, such as pseudo-Class III and skeletal Class III, is crucial for selecting the appropriate intervention. Early detection and treatment of anterior crossbites are essential to prevent complications such as gingival recession, incisal wear, and adverse growth patterns. Removable functional appliances such as chin cups are valuable tools for achieving favorable outcomes. Fixed appliances further enhance treatment efficacy by providing precise control over tooth movement and increasing the maxillary arch perimeter. Overall, a tailored approach that considers the individual patient's needs and growth potential, is key to successful orthodontic management of Class III malocclusions.
